# Temporal Control of Gelation and Polymerization Fronts Driven by an Autocatalytic Enzyme Reaction

**DOI:** 10.1002/anie.201510604

**Published:** 2016-01-06

**Authors:** Elizabeth Jee, Tamás Bánsági, Annette F. Taylor, John A. Pojman

**Affiliations:** ^1^ Department of ChemistryLouisiana State UniversityLouisianaLA70803USA; ^2^ Chemical and Biological EngineeringUniversity of SheffieldSheffieldS1 3JDUK

**Keywords:** biochemical networks, dynamic materials, frontal polymerization, gels, systems chemistry

## Abstract

Chemical systems that remain kinetically dormant until activated have numerous applications in materials science. Herein we present a method for the control of gelation that exploits an inbuilt switch: the increase in pH after an induction period in the urease‐catalyzed hydrolysis of urea was used to trigger the base‐catalyzed Michael addition of a water‐soluble trithiol to a polyethylene glycol diacrylate. The time to gelation (minutes to hours) was either preset through the initial concentrations or the reaction was initiated locally by a base, thus resulting in polymerization fronts that converted the mixture from a liquid into a gel (ca. 0.1 mm min^−1^). The rate of hydrolytic degradation of the hydrogel depended on the initial concentrations, thus resulting in a gel lifetime of hours to months. In this way, temporal programming of gelation was possible under mild conditions by using the output of an autocatalytic enzyme reaction to drive both the polymerization and subsequent degradation of a hydrogel.

There is much interest in the design of functional and adaptive polymer systems by the use of reaction networks under kinetic control.^1^ Recent strategies for the spatial and temporal control of gelation were inspired by the dissipative structures that form far from equilibrium in natural systems, such as actin filaments. Hence, gel lifetime has been controlled by tuning the timescale of competing self‐assembly and disassembly processes by using enzyme catalysts, the in situ formation of gelators, or the injection of promotors for self‐assembly and/or deactivators for self‐destruction.^2^ In these systems, gelation began immediately after the addition of the catalysts/fuel.

Many materials‐chemistry applications, such as adhesives, coatings, sealants, and injectable biomedical formulations, require an initial slow reaction followed by rapid curing. For injectable biomedical formulations, subsequent degradation of the gel for drug release is also desirable. An induction period before the rapid reaction can be introduced either through the consumption of an inhibitor that prevents the accumulation of products, for example, time‐lapse polymerization was possible in a base‐catalyzed thiol‐Michael addition reaction by the use of acid inhibitors,^3^ or as the result of an initial slow evolution of a chemical species or heat. In free‐radical polymerization, the exothermicity of the reaction can lead to an increase in the rate as the reaction progresses (thermal feedback); however, it may result in thermal runaway.^4^ Other rate‐acceleration processes are also difficult to control, such as the lag phase that occurs in supramolecular polymerization as a result of slow initial nucleation steps^5^ and the Trommsdorff–Norrish gel effect with a decrease in the rate of termination as gelation proceeds.^6^

One advantage to the presence of thermal feedback in polymerization is the ability to create cure‐on‐demand systems in which the formulation does not react until the external application of localized heating and then propagates as a constant‐velocity (cm min^−1^) polymerization front.^7^ Adhesives, for example, can be readily applied as a liquid or paste and then rapidly cured even in inaccessible locations by the propagation of the front from the initiation site.^8^ Frontal polymerization has also been used to create intricate endoskeletons in flexible materials.^9^ However, thermal frontal polymerization requires relatively thick layers and/or surfaces that are poor thermal conductors lest heat loss quench the propagation. It also involves large temperature changes (>100 °C).

Other techniques for initiating polymerization, such as irradiation with UV light, have also been used for generating fronts. Photofrontal polymerization typically requires a constant input of light for propagation.^10^ Isothermal fronts can be initiated from a polymer seed to produce gradient‐index optical materials, but these fronts are limited to free‐radical systems that exhibit the gel effect and whose polymers are soluble in their monomers.^11^

We have developed a method for time‐lapse gelation and polymerization fronts for cure‐on‐demand applications under mild conditions. Rather than exploiting intrinsic rate acceleration in the polymerization process, we used the product of an aqueous‐phase autocatalytic reaction to drive the formation of a thiol–acrylate hydrogel. The time to gelation can be controlled through the initial concentrations. Furthermore, gel lifetime can be tuned, as the gel is susceptible to hydrolytic degradation, the rate of which also depends on the initial composition.

Autocatalytic reactions are frequently exploited for the design of complex dynamic behavior in systems chemistry and synthetic biology.^12^ There are many autocatalytic systems that show induction periods and propagating fronts, including inorganic systems,^13^ self‐replicating organic reactions,^14^ and biological and biopolymeric systems, such as the propagating fronts of RNA replication^15^ and small DNA oligonucleotides.^16^ Although fascinating systems, they do not readily lend themselves to practical applications, as they either involve harsh, oxidative chemicals or cannot easily be coupled to other processes.

To create a system that operates under mild conditions, we used an autocatalytic enzyme‐catalyzed reaction: the urea–urease reaction. Urease is a well‐studied enzyme that is present in many natural systems, including various plants, soil, and microorganisms, such as the bacterium *Helicobacter pylori*, which uses the reaction to raise its local pH, thereby protecting itself against the acidic environment of the stomach. The urease‐catalyzed hydrolysis of urea has been exploited in enzyme‐based logic gates,^17^ biocement for crack healing,^18^ and chitosan gels for cell delivery;^19^ its autocatalytic nature, however, was not used in these applications.

The urea–urease reaction displays rate acceleration as a result of its bell‐shaped rate–pH curve coupled with the production of a base (Figure 1 a). If the initial pH value is low (pH≈4), a slow increase in pH occurs, followed by a rapid conversion to the high‐pH state (pH≈9), because the formation of ammonia leads to an increase in the rate of production of ammonia. The induction period of the reaction can be taken as the time for the reaction to reach pH 7 and has a well‐defined dependence on the initial concentrations of urease, urea, and acid, as well as the temperature.^20^ The reaction displays useful features inherent to autocatalytic reactions, including the ability to respond to a small amount of base with a transition from the low‐pH “off” state to the high‐pH “on” state, and the potential for oscillations and propagating pH fronts.^21^

**Figure 1:**
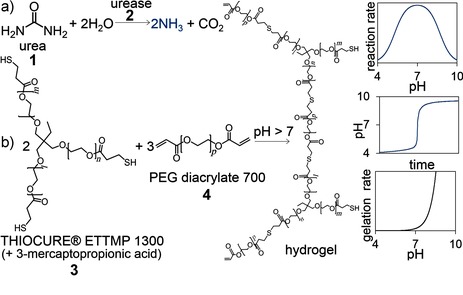
a) The reaction of urea and urease (**1**,**2**) produces a base that drives b) thiol–acrylate (**3**,**4**) gelation after an induction period. Sketched graphs show the dependence of the urea–urease reaction rate on the pH value, the resultant pH–time curve, and the dependence of the thiol–acrylate gelation rate on the pH value.

The high‐pH state of the urea–urease reaction can be used to drive a base‐catalyzed thiol‐Michael addition reaction (Figure 1 b). Thiol–acrylate chemistry has a wide range of applications, owing in part to the mild conditions the reactions need, the presence of thiols in biological systems, and the great variety of monomer options.^22^ We used water‐soluble monomers, ethoxylated trimethylolpropane tri(3‐mercaptopropionate) (Thiocure ETTMP 1300) and poly(ethylene glycol) diacrylate (PEGDA 700), to create a one‐pot aqueous‐phase system. A similar reaction was previously characterized in phosphate buffers and displayed an exponential dependence of the gelation rate on the pH value with a gelation time of seconds when the pH value was above 8 at *T*=25 °C.^23^

When a solution of urease was added to a solution of urea/ETTMP/PEGDA (Figure 2 a), the initial pH value was around 4 as a result of the small amount of 3‐mercaptopropionic acid (3‐MPA) present in ETTMP (see the Supporting Information for further details) and increased to more than 8 after an induction period (Figure 2 a). The sigmoidal characteristic of the pH–time curve was preserved with different initial concentrations (Figure 2 b). The Michael addition reaction had no discernible effect on the change in the pH value, as determined in control experiments performed with water in place of the PEGDA. Gelation took place rapidly above pH 8 and was accompanied by a cessation in motion of the magnetic stirrer.

**Figure 2:**
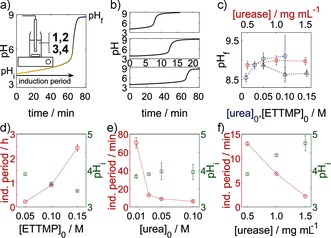
Temporal control of thiol–acrylate gelation with the urea–urease reaction. a) Typical pH–time curve with the induction period, initial pH value after mixing, and final pH value indicated. Gelation occurred rapidly above pH 8. The inset shows the experimental set‐up (see the Supporting Information). b) Series of pH–time curves with decreasing urea concentration (0.05 (top), 0.03 (middle), 0.01 m (bottom)). c) Average final pH value as a function of the initial concentrations. d–f) Induction periods and average initial pH values after mixing. The fixed initial concentrations were [urea]_0_=0.03 m, [ETTMP]_0_=0.05 m, and [urease]_0_=0.5 mg mL^−1^ (17 units mL^−1^); the ETTMP/PEGDA molar ratio was 2:3; *T*=25 °C. Standard deviations from three repeat measurements are shown.

The final pH value ranged from 8.5 to 9.5; lower values of about 7 are also possible for smaller urea concentrations (Figure 2 c).21a The final pH value was determined by the ammonia/ammonium ratio, which depended upon the initial amount of urea and the acid. Thus, the final pH value increased as the concentration of urea increased and decreased as the concentration of ETTMP or 3‐MPA increased, but remained approximately constant with changes in enzyme concentration. The initial pH value after mixing was determined by both the pH value of the ETTMP stock solution and the production of ammonia during the mixing period. Hence, the initial pH value decreased with increasing ETTMP concentration and increased with increasing urea and enzyme concentrations, even though the pH value of the ETTMP stock solution remained the same in the latter cases (Figure 2 d–f).

The induction period depended upon the rate of production of ammonia and increased with decreasing concentrations of urea and urease. The time to reach pH 7 was thus inversely correlated with the initial pH value after mixing. The trends of the dependence of the induction period on the initial concentrations agree well with those found in our earlier study without ETTMP when the pH value of the stock solution was adjusted with sulfuric or acetic acid.21a The induction period depends on the nature of the acid, as weak acids can buffer the pH change, thus reducing the reaction rate. A different acid (or base) could be added to the stock solution to tune the induction period independently of the ETTMP concentration.

Thus, the time before gelation can be controlled by tuning the initial composition of the reaction mixture with three variables: the substrate, enzyme, and acid. Reproducible induction times of several minutes to hours were observed under the conditions specified. Theoretically, induction times of months are possible in a one‐pot system, but in practice the reaction is limited by the eventual loss of enzyme activity in solution (days to weeks, see the Supporting Information). Increased enzyme stability and longer induction times are possible at lower temperatures.

An additional degree of control over the time to gelation is made possible by the ability of the reaction to support propagating pH fronts. In a thin layer (1 mm) in a petri dish, the enzyme‐catalyzed reaction was initiated locally by the addition of a base, thus giving rise to a reaction–diffusion front that converted the medium from acid (yellow) to base (blue), as visualized by the use of a pH indicator. The increase in pH locally catalyzed the Michael addition reaction, thus resulting in polymerization fronts that converted the mixture from a liquid into a gel before the corresponding induction period in a well‐stirred mixture was complete. The polymerization front was imaged by shadowgraphy (see the Supporting Information for further details).^24^ With this technique, the position of the polymerization front was visible as a dark band surrounding an expanding blue disk (Figure 3 a).

**Figure 3:**
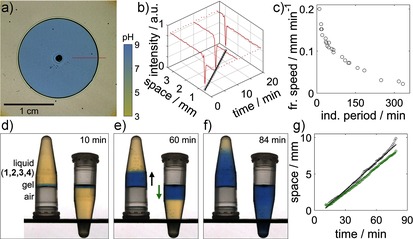
Frontal polymerization. Experiments with a pH indicator (bromothymol blue) in a–c) a thin layer in a petri dish and d–g) two 4 mL vials with identical reaction mixtures. Fronts were initiated with NaOH (pH 8). a) Image taken by shadowgraphy (see the Supporting Information) showing the position of the polymerization front (dark band) and the pH front (yellow to blue). b) Intensity profiles along a slice (red line in (a)) in images at three different times (high intensity: yellow; low intensity: blue; spike: dark band) and position of the front with time (circles with a straight‐line fit). c) Front speed in the petri dish as a function of the induction period in a well‐stirred mixture. d–f) Images of ascending and descending fronts. g) Front position as a function of time (black: ascending fronts; green: descending fronts). Component concentrations: [ETTMP]_0_=0.17 m, [PEGDA]_0_=0.24 m, [urease]=29 units mL^−1^; *T*=20 °C; [urea]=0.11 (a,b), 0.04–0.27 (c), 0.09 m (d).

The intensity profiles along a horizontal slice in images at three different times are shown in Figure 3 b. Unlike diffusive processes alone, in which a chemical becomes progressively more dilute in space, the amplitude of chemical change associated with autocatalytic fronts is constant.21a, 25 In this case it corresponds to the pH change observed in the experiments with well‐stirred mixtures. Thus, at each point in space the rate of increase in pH, the final pH value, and the rate of conversion from a liquid into a gel is the same, but there is a phase lag between points corresponding to the time at which the front passes.

Autocatalytic reaction fronts propagate with constant velocity; hence, a linear space–time plot was obtained for the polymer front (Figure 3 b). Fronts propagated with speeds that ranged from 0.02 to 0.2 mm min^−1^, depending on the initial concentrations (Figure 3 c). The velocity of autocatalytic reaction–diffusion fronts is dictated by the reaction rate and diffusion coefficient of the autocatalytic species, in this case, the base. Thus, the front speed can be related to the induction period of the well‐stirred reaction: a shorter induction period resulted in a faster front (Figure 3 c). The fronts observed in this study were slower than corresponding fronts obtained in the absence of polymerization in earlier aqueous‐phase experiments, probably as a result of the increased viscosity of the reaction mixture.21a

Convective effects can lead to front speeds that depend on the orientation of the reaction vessel and enhance mixing or even extinguish the front, thus limiting cure‐on‐demand applications.^26^ To determine whether the fronts were subject to convective effects, we compared the velocity of ascending and descending fronts in small vials. In two vials with identical reaction mixtures, a drop of NaOH (pH 8) was added to the side of the vial, which was then slowly tilted until the drop of NaOH met the surface of the liquid containing components **1**–**4**. A gel immediately formed where the base was added. One of the vials was inverted, and the thin gel layer was able to support the liquid above it (Figure 3 d). The polymerization fronts propagated with a constant velocity until the entire reaction mixture switched to a high pH value at the end of the induction period (Figure 3 d–f). Ascending fronts (Figure 3 g, black line) had a slightly higher speed than the descending fronts (Figure 3 g, green line).

Thus, the liquid can be injected into a cavity in any orientation, and a reaction can be initiated to form a plug at the open end and is then propagated as a front to cure the rest of the mixture. The coupling of the base‐catalyzed thiol‐Michael addition reaction with the autocatalytic enzyme reaction results in a new method for frontal polymerization that does not require a constant external perturbation to be maintained or involve large temperature gradients.

Considerable interest in PEG–acrylate hydrogels revolves around their use as degradable biomaterials, for example, in drug and cell delivery and as scaffolds in tissue repair.^27^ A similar PEG‐based hydrogel to the one constructed in this study was demonstrated to undergo hydrolytic degradation over the course of weeks, thus resulting in controlled drug release.^23^ More recently, a method for the pH control of the self‐assembly and disassembly of peptide hydrogels was proposed for fluidic guidance in channels and self‐erasing rapid prototyping.^28^

The thiol–acrylate hydrogels formed in this study have both an inbuilt time to gelation and an inbuilt time before complete degradation (Figure 4 a). Degradation proceeded slowly, and the mixture returned to the liquid state. Base‐catalyzed ester hydrolysis provides a convenient method for the control of gel degradation. While the time before gelation is mainly governed by the components of the urea–urease reaction, the degradation time also depends on the gel strength and hence the precursor concentrations. Gel strength was followed by dynamic rheometry. A rapid increase in the storage modulus *G′* was observed after a lag phase (Figure 4 b). With an increase in the ETTMP concentration, the maximum *G′* value increased (Figure 4 b, black and red curves), and the degradation time increased. An increase in the ETTMP concentration increases the cross‐linking density but also decreases the final pH value, thus additionally slowing the rate of base‐catalyzed hydrolysis. A decrease in urea concentration also resulted in an increase in *G′* and a slower degradation rate (Figure 4 b, red and green curves) because of the lower final pH value and higher polymer conversion associated with the longer induction time.

**Figure 4:**
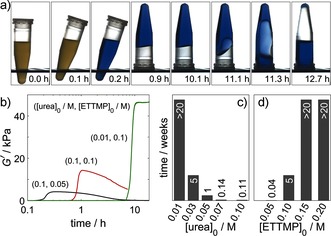
Hydrogel degradation. a) Series of images showing the return of the thiol–acrylate gel to the liquid state, in which [urea]=0.09 m, [urease]=0.85 mg mL^−1^ (29 units mL^−1^), [ETTMP]_0_=0.06 m, and [PEGDA]_0_=0.08 m. b) Dynamic rheology with storage modulus *G′* as a function of time. c,d) Time to return to the liquid state as a function of the urea (c) and ETTMP concentrations (d). Concentrations of other components: [ETTMP]_0_=0.1 m, [urea]_0_=0.03 m, [urease]=0.5 mg mL^−1^ (17 units mL^−1^); the ETTMP/PEGDA molar ratio was 2:3; *T*=23 °C.

The time for the gel to return to the liquid state varied from 5 h to over 20 weeks (Figure 4 c,d). Fast degradation times were favored by a high final pH value and low gel strength: hence, high urea and low thiol concentrations. In the examples shown, the degradation time was correlated with the induction period; however, it may be possible to independently vary these characteristic timescales through simultaneous variations in two of the control variables: enzyme, substrate, and acid.

Herein we have shown how the amplification of a chemical signal might be translated into a physical response: an autocatalytic enzyme reaction was used to drive time‐lapse gelation and frontal polymerization. The gel lifetime was also controlled through the initial concentrations of the components of the enzyme reaction and the thiol. The coupling of autocatalytic reactions with physical processes has generated pulses of precipitates,^29^ bioinspired chemomechanical devices,^30^ thiol–acrylate microparticles,^31^ and periodic nanoparticle aggregation;^32^ however, these systems involved harsh chemicals that limit their use in applications. We used an enzyme‐catalyzed reaction with a water‐soluble thiol and acrylate to create a gelation process that operates under ambient, aqueous‐phase conditions.

Our system does not require radical initiators or a high temperature but operates on the basis of an inbuilt pH switch. Other autocatalytic enzyme reactions, such as the glucose–oxidase reaction, involve base‐to‐acid switches that might be used in conjunction with acid‐catalyzed polymerization.^33^ This systems‐chemistry approach to transient gelation has numerous attractive features for bioinspired, biocompatible materials applications.

## Supporting information

As a service to our authors and readers, this journal provides supporting information supplied by the authors. Such materials are peer reviewed and may be re‐organized for online delivery, but are not copy‐edited or typeset. Technical support issues arising from supporting information (other than missing files) should be addressed to the authors.

Supplementary
