# Supplementary material for: Temporal Control of Gelation and Polymerization Fronts Driven by an Autocatalytic Enzyme Reaction
Source: Angew Chem Int Ed Engl. 2016 Jan 6;55(6):2127–31. doi: 10.1002/anie.201510604 (PMC4755207; doi:10.1002/anie.201510604)
Supplement: Supplementary file 1 — Supplementary [file ANIE-55-2127-s001.pdf]

Angewandte Chemie
Eine Zeitschrift der Gesellschaft Deutscher Chemiker

Supporting Information

Temporal Control of Gelation and Polymerization Fronts Driven by an Autocatalytic Enzyme Reaction

Elizabeth Jee, Tamás Bánsági, Jr., Annette F. Taylor, and John A. Pojman**

anie_201510604_sm_miscellaneous_information.pdf

Supporting Information

1. Materials

The urease (U1500, type III from Jack Bean, 34310 units/g solid) and poly(ethylene glycol) diacrylate (PEGDA, Mn = 700 g/mol, ρ = 1.12 g/ml) were purchased from Sigma Aldrich. Extra pure (>98%) urea pearls were obtained from Acros Organics. Universal indicator and bromothymol blue (pKa = 7.1) were used to visualize pH changes in front experiments. Ethoxylated trimethylolpropane tri(3-mercaptopropionate) (THIOCURE® ETTMP 1300, Mn (approx.) = 1300 g/mol, ρ = 1.15 g/ml)) was acquired from Bruno Bock Chemicals. All chemicals were used as received (radical inhibitors were not removed from PEGDA).

THIOCURE® ETTMP contains < 1% 3-mercaptopropionic acid 3-MPA (pKa = 4.34) as impurity. The acid number from five separate batches was (according to the supplier): 3.7 ± 0.3 i.e. less than a 10% standard deviation in the initial acid concentration (and a potential variation of pH of the stock solutions of ± 0.04 between batches). The concentration of ETTMP in the stock solutions was determined from the mass and approx. Mn noted above. The average pH of three different solutions of a particular concentration of ETTMP in water was found to be reproducible: 3.14 ± 0.01 (0.05 M); 2.91 ± 0.02 (0.1 M) and 2.73 ± 0.02 (0.15 M) with a standard deviation of no more than 0.7%.

The enzyme concentration in the stock solution was determined from the mass in mg added to 25 mL of water. The activity in units/ mL was determined from the activity of 34310 units/ g noted above. There is a slow loss of activity of enzyme in solution therefore stock solutions were used freshly prepared or kept in a refrigerator between uses.[1] In trials that compared the activity of enzyme (32 units mg^{-1}) in buffer at different temperatures the half-life (time for the enzyme activity to decay to half its initial value) was of the order of 13 days at 4 °C and 3 days at 25 °C.[2] In other trials with urease from different sources in alternative buffers the half-lives at 4 °C were 19 and 21 days.[3] Hence the rate of decay may depend on the source of urease and storage solution and is of the order of weeks when refrigerated.

2. Induction time and pH measurements in well-stirred experiments

Vernier pH probes and Logger Lite software were used to obtain pH-time data. Two 25 mL stock solutions were prepared: the first solution contained urea, ETTMP, PEGDA, and water; the second solution contained urease in water. Then 8 mL of urea solution was dispensed into a 50 mL beaker equipped with a stir bar (5/8” × 5/16”) and a pH probe on a magnetic stirrer. The reaction was initiated by the addition of 8 mL of the urease solution. The concentrations reported were the final concentrations produced after mixing. The experiments were performed at room temperature (25 °C).

The average and standard deviation of the induction time, initial pH (after mixing), and final pH were recorded from three repeat measurements from the stock solutions. The induction periods were determined as the time to reach pH 7 and were reproducible (5% standard deviation on average) for the specified set of conditions. Gelation occurred rapidly above pH 7 and was accompanied by the loss of motion of the magnetic stirrer bar, several minutes after the induction period.

The standard deviation in initial pH after mixing in the urea-urease experiments varied between 0.3 and 6%. Sources of error include slow response time of the pH electrode and variations in the rate of ammonia production during the mixing process. Similar standard deviations were observed in the measurements of the final pH (1 – 6%).

Since the reaction produces the volatile gas ammonia, the induction times and final pH will depend on whether or not there is an open air interface and the rate at which ammonia is allowed to escape into the atmosphere. In trials comparing open and sealed reactors with the same initial solution concentrations, the induction times were found to be 13.6 minutes and 13.9 minutes i.e. 2% longer in open vials because the pH increased more slowly when ammonia was lost to the atmosphere.

3. Shadowgraphy

The purpose of the shadowgraph experiments was to show that the polymerization front was directly coupled to the enzymatic reaction-diffusion front that converts the medium from acid to base and travels with constant velocity in the absence of polymerization (as discussed in our earlier work).

Shadowgraphs and transmission images of polymerization fronts propagating horizontally in 10 cm diameter Petri dishes (Corning) with the dish placed inside the upside-down lid were taken with a PixeLink CCD camera connected to a computer (Figure S1a). For illumination, a MiniSun A4 LED light pad and a white LED (diameter: 5 mm, epoxy dome removed) with a 100 mm diameter, 200 mm focal length plano convex condenser lens (Edmund Optics) placed in the light path just underneath the dish holder stage were used alternately. Time elapsed between corresponding RGB images and shadowgraphs was 2 seconds. Universal indicator was used to visualize the basic front. Fronts were initiated externally by placing 2 µl urea-urease product solution containing ETTMP monomer but no PEGDA through a 0.5 mm hole at the center of the bottom dish, which was used as the cover. The initiating solution has a similar composition and pH to the final gel product in order to minimise transport of species arising from mixing effects rather than reaction.

In the control experiment without PEGDA, the pH front can be clearly visualized with universal indicator as a green circle propagating into the orange acidic medium (Figure S1b, left half of image). Switching to the shadowgraph image, no change in the gradient of the refractive index was discernible with the pH front (Figure S1b, right half of image). However, upon inclusion of the acrylate, polymerization fronts were observed propagating with constant velocity that depended on the initial concentrations of urea and urease (Figure S1c). The shadowgraph images showed a bright and dark band around the circular front (Figure S1d-f) indicative of the change of refractive index associated with the polymerization.

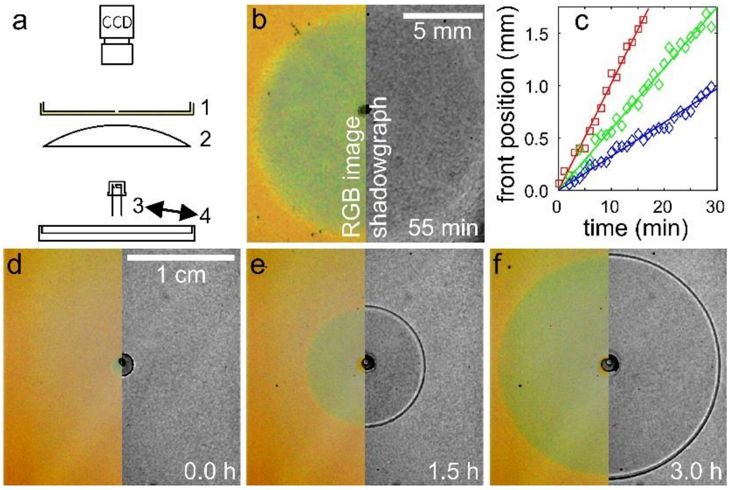

Figure S1. Experimental set up for shadowgraph experiments. 1 = petri-dish and inverted lid (hole in the petri dish for initiation of the front), 2 = lens, 3 = LED light source for shadowgraph and 4 = light pad for RGB image. (b) Control experiment where left half shows RGB image and right half shows shadowgraph image with [PEGDA] = 0, [ETTMP] = 0.17 M, [urea]= 0.05 M. (c) front position in time where red: [ETTMP] = 0.17 M, [PEGDA] = 0.24 M, [urea] = 0.05 M, slope:

0.10 mm/min; green: same as in (d - f), slope: 0.06 mm/min; blue: [ETTMP] = 0.17 M, [PEGDA] = 0.24 M, 0.01 M [urea]; slope: 0.03 mm/min. (d - f): [ETTMP] = 0.17 M, [PEGDA] = 0.24 M, 0.02 M [urea]; Time elapsed between the shadowgraphs and the corresponding RGB images: 2 sec. In all experiments: layer depth = 1 mm; urease = 36 units/ml, T = 20 °C; front is visualized using 6 v/v% universal indicator where ahead of the front is orange unreacted low pH (~3.5 - 5) and behind the front green reacted high pH (≥8). The yellow ring that can be seen in between the propagating pH front and unreacted area is ~pH 6-7.

4. Rheology

Rheological measurements were conducted on a TA AR2000ex rheometer. Time sweep measurements were conducted over 24 hours to monitor the change in storage and loss moduli as the sample was sheared. The angular frequency (0.628 rad/s), strain (1%), and temperature (23 ºC) were kept constant. Two separate solutions were made for each test, urea/thiol/acrylate and urease. After mixing the two solutions, 1.50 mL was applied to the Peltier plate before lowering the 40 mm steel parallel plate to a starting gap of 1.000 mm.

5. Degradation experiments

Experiments were performed with components 1 – 4 mixed and then added to 4 ml sealed vials. One group involved a set of urea variations (0.01 M - 0.10 M) with constant urease (0.5 mg/mL) and constant ETTMP (0.10 M). The other group had ETTMP variations (0.05 M - 0.20 M) with constant urease (0.5 mg/mL) and urea (0.03 M). All trials had 1:1.5 molar ratios of ETTMP:PEGDA. So as ETTMP concentrations changed, PEGDA concentrations were altered accordingly. Samples were determined to be gelled when an air bubble could no longer move through the polymer upon inversion of the vial. Conversely, after gelation was established, degradation time was recorded when the air bubble could once again move freely upon inversion. The timer was started upon mixing of the solutions.

References

[1] B. Krajewska, *J. Mol. Catal. B: Enzym.* **2009**, *59*, 22-40.

[2] B. Krajewska, M. Leszko, W. Zaborska, *J. Chem. Technol. Biotechnol.* **1990**, *48*, 337-350.

[3] aS. Kumar, A. Dwevedi, A. M. Kayastha, *J. Mol. Catal. B: Enzym.* **2009**, *58*, 138-145; bR. C. Reddy K, P. K. Srivastava, P. M. Dey, A. M. Kayastha, *Biotechnol. Appl. Biochem.* **2004**, *39*, 323-327.
